# An evolutionary analysis of new energy and industry policy tools in China based on large-scale policy topic modeling

**DOI:** 10.1371/journal.pone.0252502

**Published:** 2021-05-28

**Authors:** Qiqing Wang, Cunbin Li

**Affiliations:** 1 School of Economics and Management, North China Electric Power University, Beijing, PR China; 2 Beijing Key Laboratory of New Energy and Low-Carbon Development, North China Electric Power University, Beijing, PR China; Shenzhen University, CHINA

## Abstract

This study investigates the evolution of provincial new energy policies and industries of China using a topic modeling approach. To this end, six out of 31 provinces in China are first selected as research samples, central and provincial new energy policies in the period of 2010 to 2019 are collected to establish a text corpus with 23, 674 documents. Then, the policy corpus is fed to two different topic models, one is the Latent Dirichlet Allocation for modeling static policy topics, another is the Dynamic Topic Model for extracting topics over time. Finally, the obtained topics are mapped into policy tools for comparisons. The dynamic policy topics are further analyzed with the panel data from provincial new energy industries. The results show that the provincial new energy policies moved to different tracks after about 2014 due to the regional conditions such as the economy and CO_2_ emission intensity. Underdeveloped provinces tend to use environment-oriented tools to regulate and control CO_2_ emissions, while developed regions employ the more balanced policy mix for improving new energy vehicles and other industries. Widespread hysteretic effects are revealed during the correlation analysis of the policy topics and new energy capacity.

## 1. Introduction

**Fig 1:**
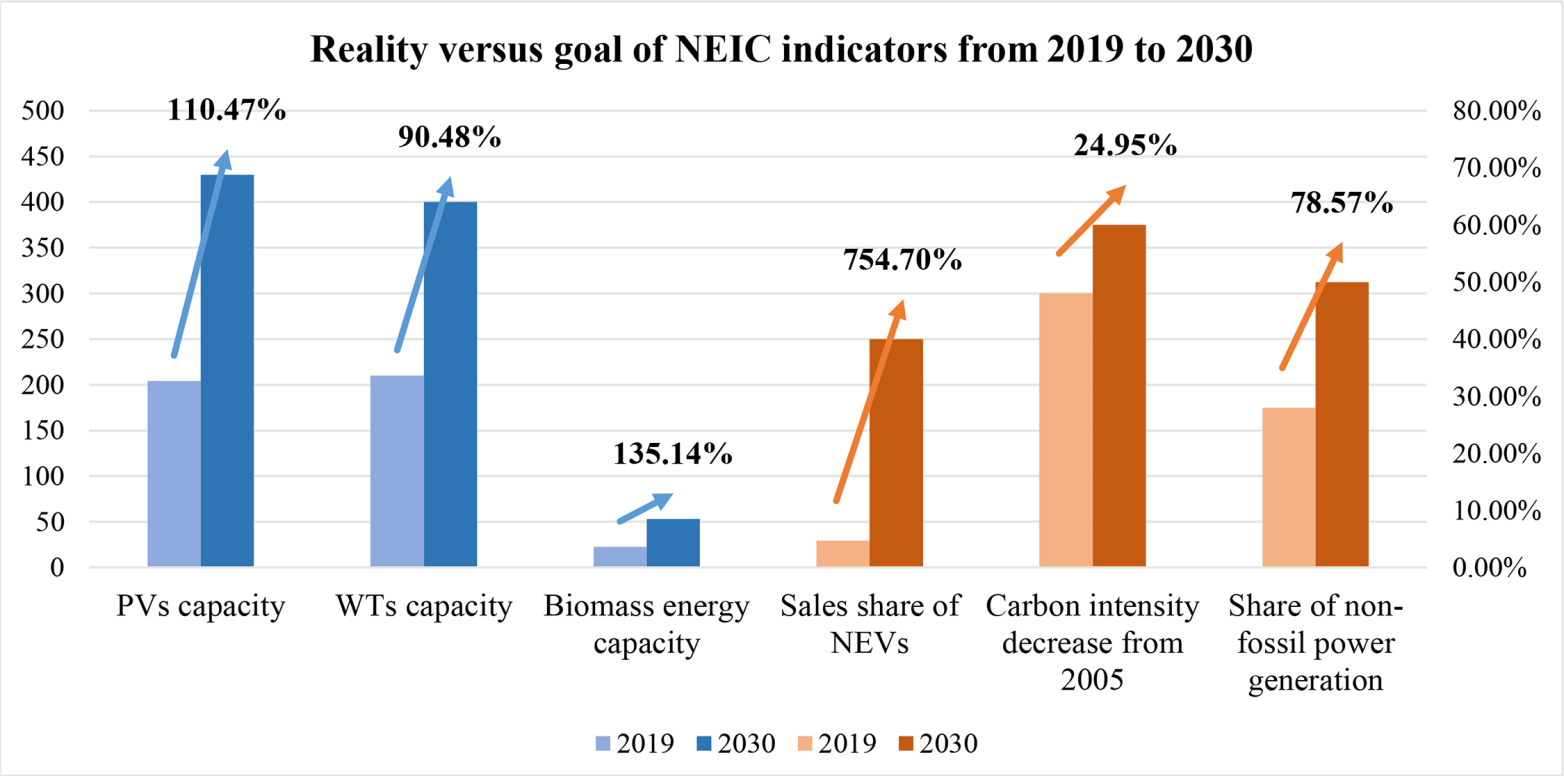
Primary NEI-related goals of China in 2030. Data sources: The data in 2019 are from the National Bureau of Statistics and NEA.

With a series of incentive new energy policies (NEPs) issued by governments, the past decade has witnessed the rapid development of new energy industries (NEIs) in China, especially the photovoltaic and wind power industry. According to the National Energy Administration (NEA) of China, the capacity of renewable energy sources (RESs) in 2019 reached about 794 million kilowatts, of which the total capacity of photovoltaics(PVs) and wind turbines (WTs) accounted for more than half [1]. Fig 1 displays several ambitious NEI-related goals in 2030 summarized from some crucial NEPs like the prospects of photovoltaic development in China in 2050 [2] and the revolutionary strategy of energy production and consumption [3]. Chinese governments have implemented the new energy industry (NEI) development as a long-term strategic plan.

However, challenges and uncertainties still exist in the NEI of China. Despite some tough goals shown in Fig 1 like increasing sales of NEVs by more than seven times in ten years, China has the largest CO_2_ emission globally with about a 2.3 percent increase in 2018, posing great challenges for future domestic energy conservation and emission reduction [4]. Moreover, China is at the turning point of the 13th Five Year Plan and the 14th Five Year Plan, changes in the environmental and economic policies can also influence the NEI development. Therefore, it is of great significance to identify the challenges of NEIs by figuring out the interaction of the new energy industry and policy.

Much literature has investigated new energy industry and policy, some works incline to study the NEPs or NEIs separately. On the NEP side, policy documents of 40 years are reviewed for investigating the sustainable energy transition and evolution that derive from the policy mix adopted by the Chinese government [5]. Factors affecting the diffusion of China’s financial subsidy policy for NEVs are investigated in [6]. Technology, economy, energy vulnerability, global effects, and human well-being are considered when making renewable energy policy plans by the MCDM method [7]. The heterogeneous preferences of the public can also impact the implementation of solar energy policies and electric vehicle policies [8,9]. The chief drivers of ecological footprint and economic growth are studied from 15 highest emitting countries [10]. Multiple panel data estimators are employed to demonstrate that natural resource rent, renewable energy, and urbanization can decrease ecological footprint [11]. On the NEI side, shaping and driving factors to the development of renewable energy industries in China are identified in [12]. Energy-change impacts on the sustainability of small and medium-sized enterprises are analyzed with a dynamic method that examines interactions between variables from a holistic, real-world perspective [13]. Higher economic growth, rising unemployment, and government debt are proved to be helpful to renewable energy generation [14].

Other research works are more comprehensive and focus on how NEPs can affect NEIs. For instance, policy portfolios including both market creation and expansion policies are proved to be effective at developing solar photovoltaic markets [15]. The efficiency evaluation of three carbon emissions policies in Indonesia shows that a carbon tax and emissions trading scheme can promote substitution towards renewable energy [16]. Regression analysis is used to explore the green innovation effect under the implementation of three environmental regulations [17]. The role of institutional quality in CO_2_ emissions is investigated via an autoregressive distributed lag model [18].

In terms of analytical methods, quantitative policy analysis has become more popular in recent years. Generally, the quantitative method can be classified into two categories, i.e., indirect and direct. For the indirect one, policies are often evaluated by measuring their impacts on the economy, industry, and environment. For example, the effect of renewable energy consumption and trade policy of the US is evaluated from the policy impact that exerts on environmental degradation [19]. An econometric model is used to assess market-pull policies in promoting net renewable capacity [20]. The effectiveness of China’s carbon market policy is evaluated with a panel data based difference-in-differences model [21,22].

The direct method for quantitative policy analysis focuses on discovering the characteristics and patterns from a large number of policies and related texts. Policy documents are often studied by text analysis approaches. Korean nuclear policy texts released in 2003 to 2016 are investigated with LSA [23], publications and articles regarding Russia’s renewable energy are categorized with big data analysis [24], official speeches on trade policies are examined by statistical text mining and LDA method [25]. Massive abstracts of papers on neighborhood sustainability are studied and discussed by clustering methods at both geographical and temporal levels [26]. Moreover, social media texts such as comments and posts can be used as materials for policy analysis, e.g., the Twitter debate about monetary policies of the U.S. is analyzed via combined computational text analysis [27]. Topics and sentiments are identified from Twitter data to explore public attitudes and factors to a transit network [28].

Among all the direct methods mentioned above, topic models like the LSA and LDA are most commonly used due to the ability to automatically process massive documents in a very short time. However, as an unsupervised topic model that can reflect the continuous and dynamic evolutional characteristics of documents, the dynamic topic model (DTM) is also adopted by many researchers. For example, a time-varying metadata-enabled DTM is proposed for capturing massive inherent dynamic features of a large Weibo dataset [29], massive US patent documents on disease management are analyzed using the DTM to determine the focuses and trends of protected technological innovations [30]. Nevertheless, although text mining technology has become more and more popular in sociology, topic models are hardly used in policy text analysis, especially in the NEPs.

Therefore, in order to explore the discrepancy of provincial NEPs from multiple perspectives like policy tool usage, policy evolution, and the role of policies in NEIs, topic models and text mining methods are used to analyze large-scale NEI-related policy documents from several representative provinces in China. The primary contributions of this paper are threefold:

1. An analytical framework based on topic models is proposed for extracting both static and dynamic policy topics from large-scale provincial new energy policies including legislation, regulation, and other official documents in the period of 2010 to 2019.
2. The extracted new energy policy topics are mapped to the supply-oriented, demand-oriented, and environment-oriented policy tools, which are used to compare the shifting focus in provincial new energy policies.
3. The interaction between provincial new energy policies and industries is compared by the correlation analysis of time-varying new energy policy topics and panel data.

This paper is organized as follows: Section 2 illustrates the collection of NEP data from six sampled provinces. Section 3 introduces the analytical framework for new energy policy and industry using topic models. Section 4 demonstrates the provincial NEP topic modeling results, which are compared and analyzed from multiple perspectives. The study is concluded in Section 5.

## 2. Data collection

In this section, both NEP-related text data and NEI-related panel data are collected from several representative provinces that are sampled from different regions of China.

### 2.1 Province selection by new energy performance

Firstly, several quantitative indicators listed below are used as the sampling criteria to evaluate the comprehensive NEI performance of 31 provinces in China, among them are:

1. Regional GDP (C1): This is the most common economic index, provinces with high GDP prefer to develop the NEI [31].
2. Electricity consumption (C2): This can be used as a supplement to the GDP indicator, it reflects regional industrial development and energy consumption. The proportion of thermal power generation in China is about 70%, a province with more electricity consumption has more potentials to replace existing thermal power consumption with new energy power.
3. PVs capacity (C3): The volume of PVs capacity can directly reflect how much local government attaches importance to NEIs.
4. WTs capacity (C4): Since the location advantages of provinces are different from each other, both PVs and WTs capacity are considered. Notably, other new energy resources such as biomass energy and geothermal energy are not included in the sampling criteria for their little disparity in different provinces.
5. Number of NESTCs (C5): The number of new energy science and technology companies engaged in the NEI of China can measure the regional NEI development as well as the governmental concerns on the NEI.
6. Number of UHV projects (C6): The ultra-high voltage (UHV) technology capable of transmitting electric power in long-distance can alleviate the impact of reverse energy distribution in China. Affected by the COVID-19, Chinese governments have vigorously supported the construction of UHV projects this year for the sake of restoring the economy. Therefore, in the future, those provinces with UHV projects would have more advantages in both new energy consumption and production [32].

The NEI performance of each province is measured with a scoring value obtained by normalizing and summing factual data of C1 to C6 in 2019 (S1 Table). Since the geographical and economic zones of China in addition to the criteria also play an important role in the sample selection, seven provinces are chosen as the candidates according to their high NEI performance scores and locations, as shown in Table 1.

**Table 1:** Evaluation results of representative provinces.

Provinces	Geographical and economic zones	C1	C2	C3	C4	C5	C6	Scores
**Guangdong**	South China/Pearl River Delta	1.00	1.00	0.36	0.15	1.00	0.00	3.50
**Jiangsu**	East China/Yangtze River Delta	0.92	0.97	0.92	0.35	0.71	0.60	4.46
**Shandong**	East China/Bohai Economic Rim	0.65	0.96	1.00	0.45	0.63	0.60	4.29
**Inner Mongolia**	North China	0.15	0.53	0.66	1.00	0.09	1.00	3.42
**Sichuan**	Southwest	0.42	0.38	0.09	0.11	0.13	0.40	1.53
Liaoning	Northeast/Bohai Economic Rim	0.22	0.36	0.19	0.28	0.23	0.00	1.27
**Sinkiang**	Northwest	0.11	0.33	0.66	0.65	0.07	0.20	2.01

Since Shandong and Liaoning provinces are both in the Bohai Economic Rim and the former one gets a much larger score than the latter, six provinces in Table 1 except for Liaoning are finally selected as research samples.

### 2.2 Text and panel datasets construction

All the NEI-related policies, regulations, and other official documents in the period of 2010 to 2019 are collected from the central government and sample provinces to build a corpus for text mining and topic modeling. The NEP text is primarily from two websites, one is the *pkulaw* (www.pkulaw.cn), which incorporates China’s national-level and local-level policy documents, another is *Bailu Thinktank* (www.bailuzhiku.com), a big data platform for displaying information released by government agencies. By setting search keywords including “new energy”, “renewable energy”, “charging pole”, “electric vehicle”, “photovoltaic”, “wind power”, “biomass energy”, etc., 23, 674 documents in Chinese are collected from the sites. Then the raw text data is cleaned by Python tools like *re* [33] and NLTK [34], the documents are segmented into words by *Jieba* [35]. Finally, a NEP corpus composed of national and provincial policy documents is established.

**Fig 2:**
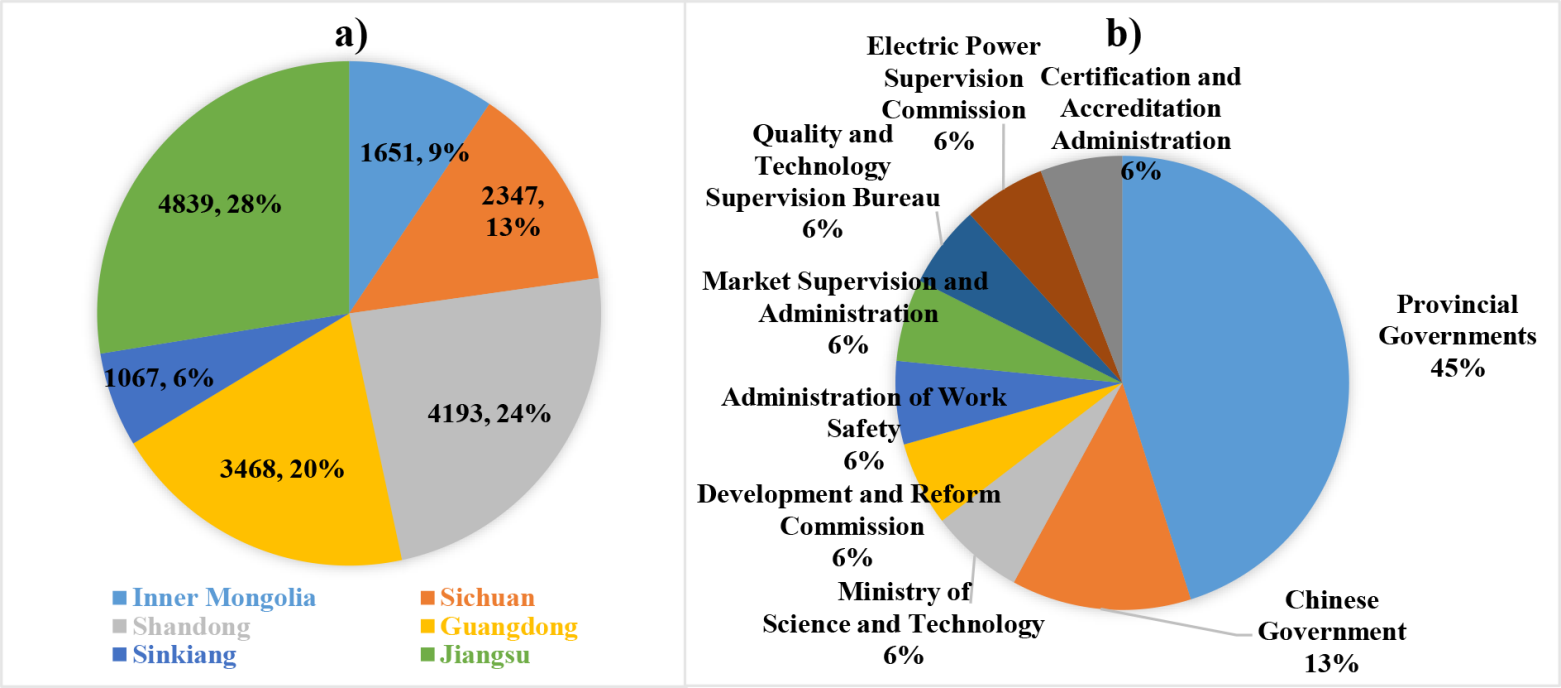
The proportion of NEPs issued by provinces and institutions: a) provincial distribution; b) institutional distribution. Subgraph b) only displays organizations that have published more than 500 NEP documents.

Fig 2 shows the NEP corpus distribution in different provinces and institutions. It can be seen from subgraph a) that Jiangsu, Guangdong, Shandong get the most policy documents, while Sinkiang the least. In subgraph b), most policies are published by national agencies due to the immaturity of domestic NEI associations or organizations, primary policy publishing bodies include location and central governments, Ministry of Science and Technology, Development and Reform Commission. Moreover, the diversity of policymakers demonstrates that NEI development has covered many other fields such as transportation, agriculture, economy, electricity, etc.

Additionally, panel data on several provincial NEI indicators from 2010 to 2019 is also collected, as shown in Table 2. The CO_2_ emission of each province per year is calculated by Formula (1) [36].

**Table 2:** NEP corpus and panel data collected from six sample provinces.

Data Types	Attributes	Description
Text corpus	Title	Policy document title
	Publishing time	Policy release time
	Content	Policy content
Panel data	GDP	Provincial GDP (a hundred million yuan)
	CO2 emissions	Provincial CO2 emissions (10, 000 tons)
	WTs capacity	The Capacity of WTs (billion kilowatts)
	PVs capacity	The Capacity of PVs (billion kilowatts)

$$E_{CO_{2}}=\sum_{i=1}^{n}F_{i}\times c_{i}\times \delta_{i}\times \eta_{i}\times \frac{44}{12}$$ (1)

Where *F*_*i*_ represents the physical amount of fuels and gas consumed by a province (*i* = 1,2,…,*n*), *c*_*i*_ is the conversion coefficient of fuel to standard coal (tce), *δ*_*i*_ is carbon emission factor (tC/tce), *η*_*i*_ is the carbon oxidation rate. The data is derived from the China Energy Statistical Yearbook, including terminal energy consumption types and quantities of each province, and standard coal conversion coefficients of various fuels [37].

## 3. Methodology

Based on the collected datasets, a comprehensive analytical framework is proposed in this section for comparing provincial policies and their shifting focal points.

### 3.1 Analytical framework

**Fig 3:**
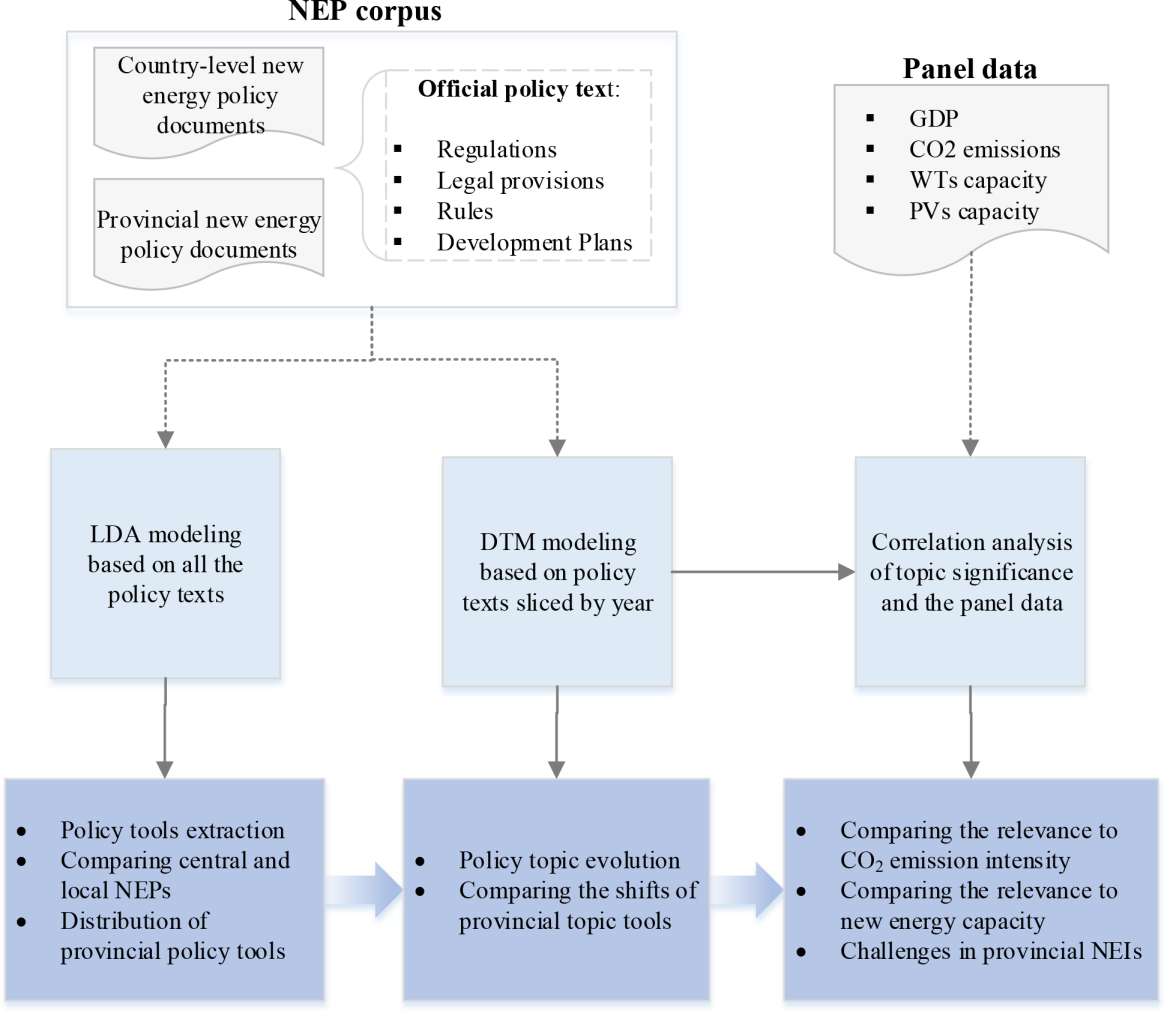
The topic model-based framework for analyzing NEPs and NEIs.

In order to extract well-organized information from the large-scale NEP text corpus, unsupervised topic models that enable extracting hidden topics in texts are used to analyze NEPs, as shown in Fig 3. Firstly, the whole NEP corpus is fed to the static LDA topic model for extracting topics that are mapped into demand-oriented, supply-oriented, and environment-oriented policy tools. Then, the NEP corpus is sliced into several sub-corpora by year. Those corpora are input to the DTM for modeling policy topics over time, thus the evolution of provincial policy tools is analyzed. Finally, several representative NEP topics are used for correlation analysis with the NEI panel data in order to further compare provincial policy characteristics and discover NEI challenges.

### 3.2 Topic models

**Fig 4:**
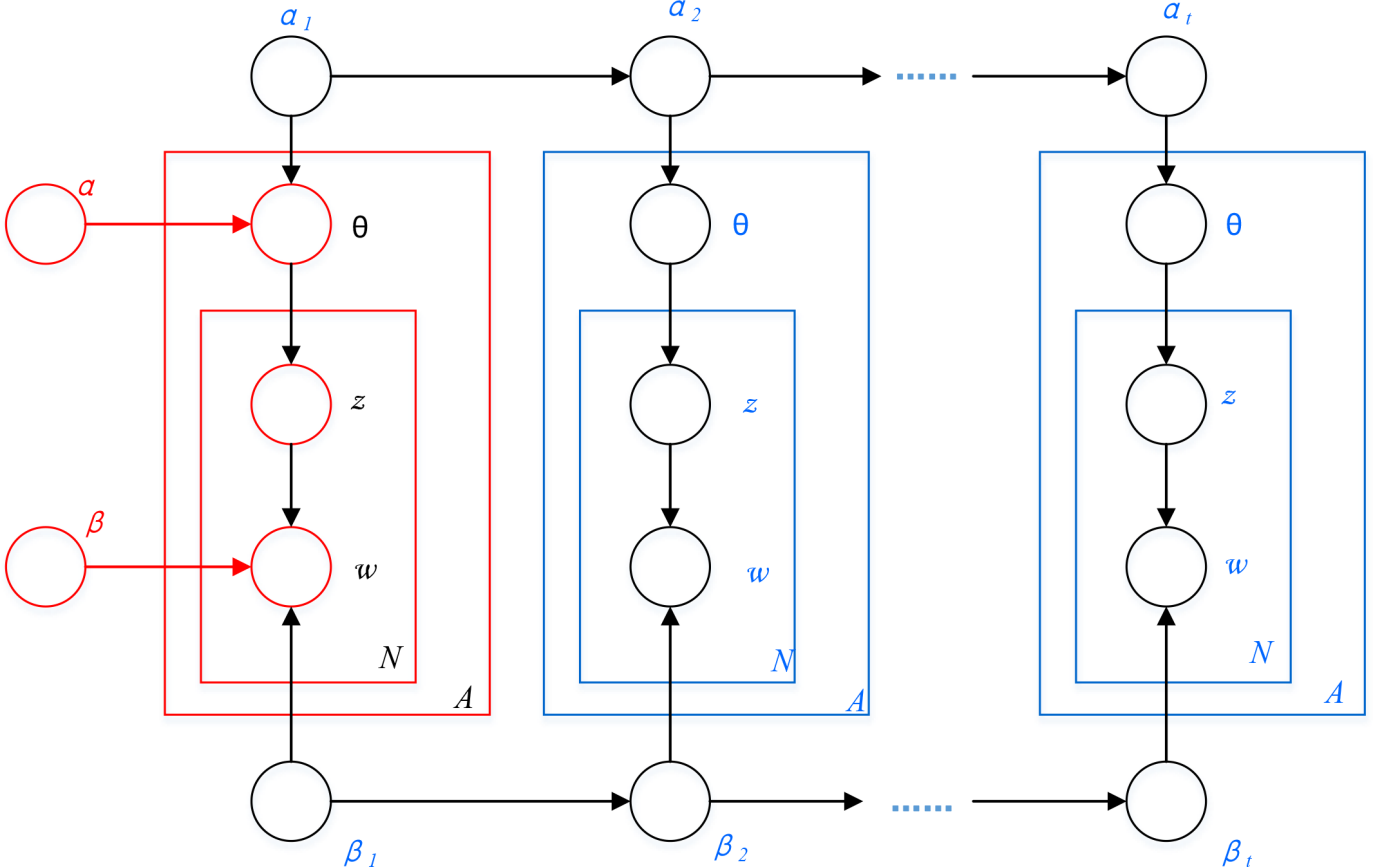
Modeling structure of LDA and DTM.

Due to the mature mathematical theory and unsupervised advantages, topic models like the Latent Dirichlet Allocation (LDA) [38] and Dynamic Topic Model (DTM) [39] have been widely applied in various fields such as biology, policy analysis, linguistics, etc. Fig 4 illustrates the difference between LDA and DTM in modeling structure. DTM (blue box) can be roughly regarded as unfolding an LDA (red box) model on *t* time steps, both of them share a similar assumption for generating documents. For example, LDA model assumes that each document *w* is produced by several sampling steps:

- Choose *N* ~ *Poisson*(*ξ*)
- Choose θ ~ *Dir*(*α*)
- For each of the *N* words *w*_*n*_:
  - Choose a topic *z*_*n*_ ~ *Multinomial*(*θ*).
  - Choose a word *w*_*n*_ from *p*(*w*_*n*_|*z*_*n*_, *β*), a multinomial probability conditioned on the topic *z*_*n*_.

In spite of the structure discrepancy between LDA and DTM, these two models are different in whether words in a corpus are hypothesized exchangeable. LDA is an exchangeable topic model where documents are assumed to be independently drawn from a mixture of multinomial, whereas a DTM model captures the evolution of topics in a sequentially organized corpus. Although LDA is even more commonly used than DTM, the latter topic model excels in automatically creating time-varying topics with bags of words that co-occur in documents according to certain patterns.

The topic models are trained by text documents that are segmented into word sequences and parameters like *θ* and topic-word distribution are learned by inference methods including maximum likelihood estimation and Gibbs sampling [38]. In the proposed analytical framework, LDA is used as a static topic modeling method for discovering characteristics of the NEPs, while DTM is applied to the time-varying NEP corpus to explore the changes of new energy policy topics over time.

## 4. Results and discussion

In this section, the proposed analytical framework is applied to the collected datasets, different topic models are used to extract new energy policy topics for comprehensive comparison among the sample provinces.

### 4.1 Comparison by static NEP topics

#### 4.1.1 Policy tool categorization by topics

**Fig 5:**
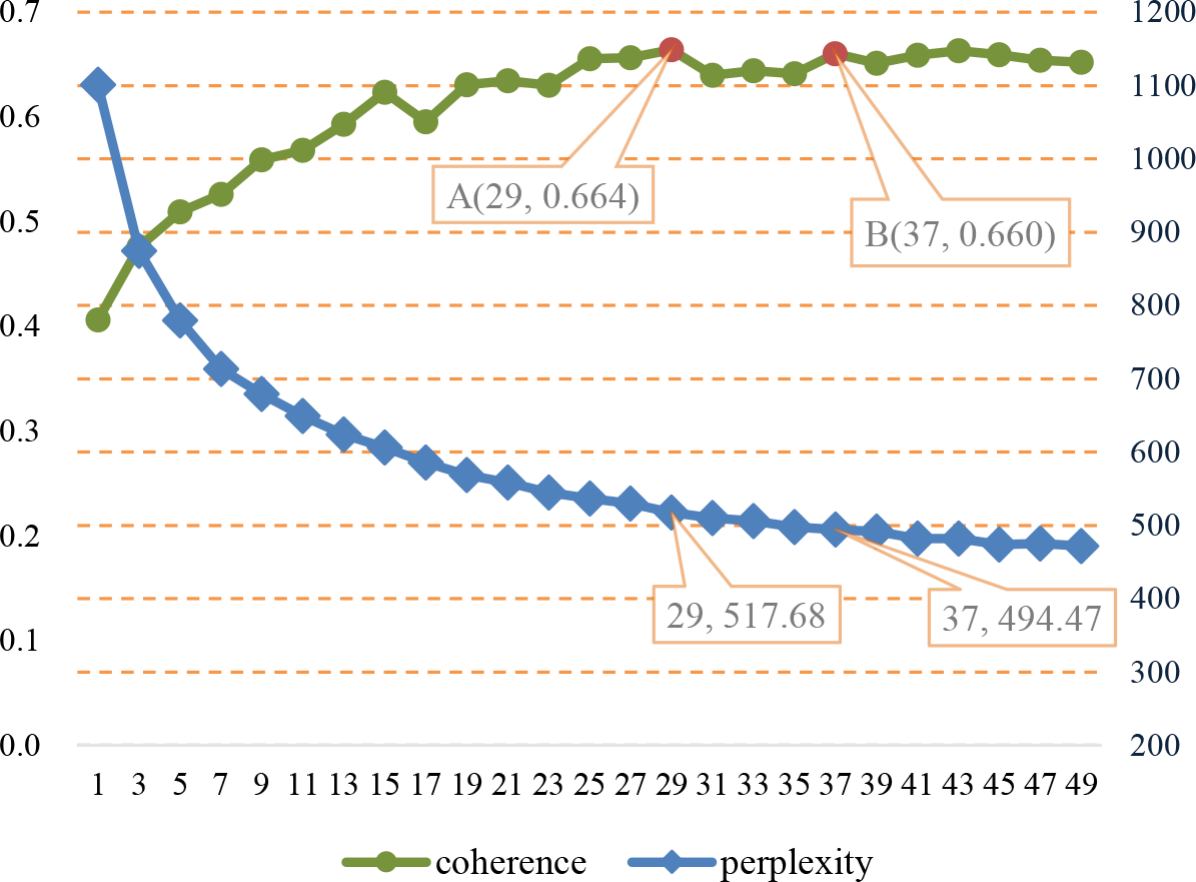
Evaluation results of LDA models with different number of topics.

The number of topics is one of the most important hyper-parameters of an LDA model capable of extracting policy tools from NEP texts. In this study, LDA models with different topic numbers (*T* = 1,3,…,47,49) are implemented by the python module *genism* [40] and evaluated by the perplexity and coherence metrics, which can measure the independence and interpretability of topics. Using the whole NEP corpus as the input of LDA models, Fig 5 shows the evaluation results of topic perplexity and coherence under different topic numbers. Since coherence-oriented topics in an LDA model are proved to be more interpretable than the perplexity-oriented ones [41], *T* = 29 corresponding to point A(29, 0.664) in Fig 5 with the highest coherence value is selected as the topic number for modeling policy topics.

**Fig 6:**
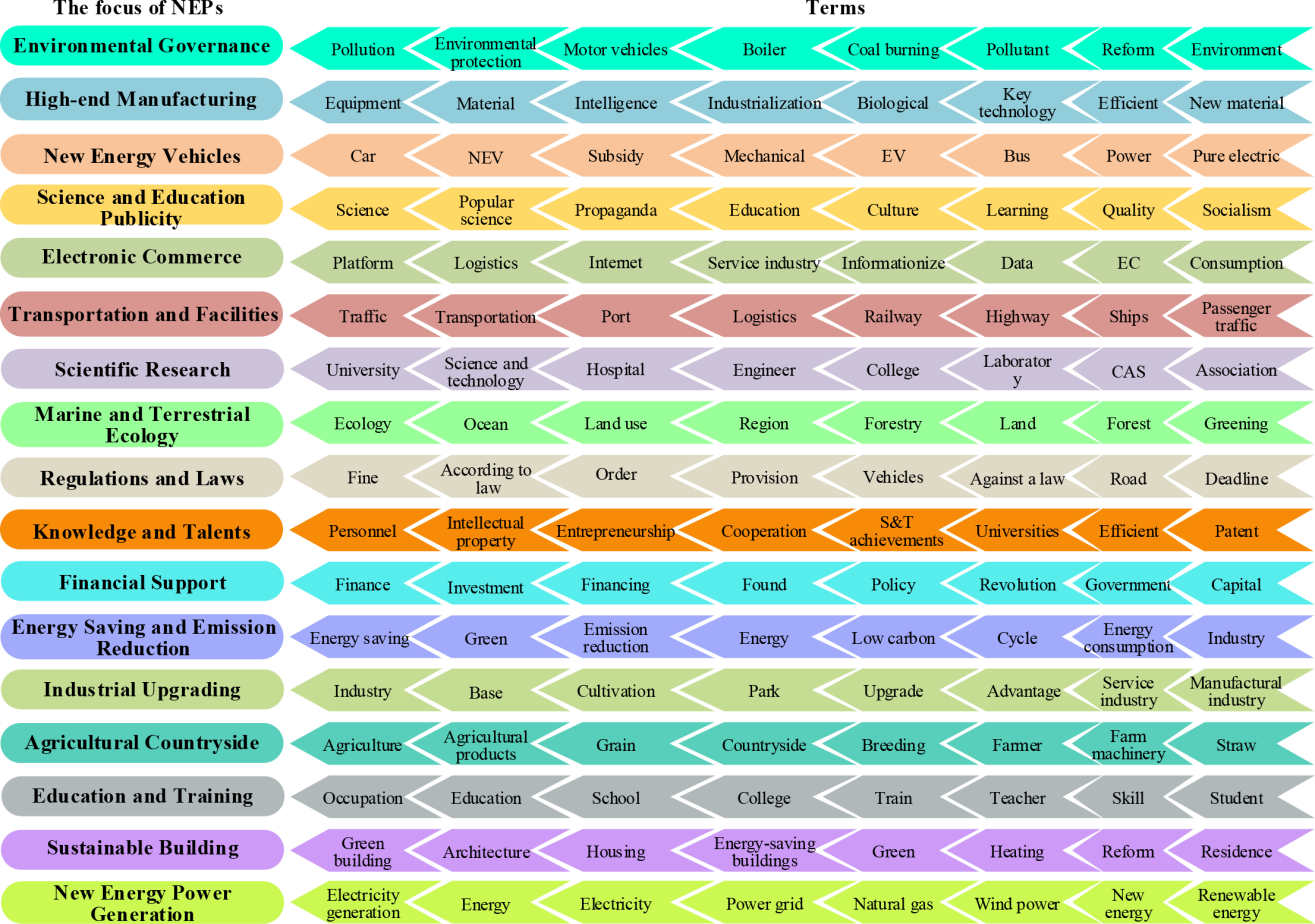
Typical NEI-related topics and top-eight terms derive from the LDA model.

Fig 6 displays some typical topics extracted by the LDA model (S2 Table), topic names in the left column are determined by comprehending the top terms in the right column. Therefore, the NEP documents released in the last decade have covered multiple fields such as environment, manufacturing, automotive industry, science and education, transportation, finance, agriculture, and construction industry, etc. Focal topics like “environment governance”, “high-end manufacturing”, and “transportation and facilities” appear in the NEP because the aim of building NEIs is to protect the environment by replacing fossil energy with solar and wind power. However, it seems that the focal topic “agricultural and countryside” that emphasizes agriculture, farmers, and countryside has little to do with NEIs, but this can be explained by the prevailing implementation of energy projects like PV and biomass generation in many villages of China.

Moreover, joint analysis can be applied in the topic-term distribution by incorporating policy instruments, which are important for understanding policymakers’ preference for new energy policies. According to the top 20 terms contained in each topic, 29 topics obtained from the NEP corpus are classified into three types of policy instruments [42], among them are:

1. Supply-oriented tools: These tools concentrate on providing human financial and material resources for the NEI. Topics that belong to the supply-oriented instrument in Fig 6 are “High-end Manufacturing”, “Scientific Research”, “Knowledge and Talents”, and “Financial Support”.
2. Demand-oriented tools: In the NEI of China, such tools are designed to stimulate industrial demand or remove barriers from the demand side. For instance, among topics of demand-oriented tools, “Sustainable Building” focuses on promoting renewable energy substitution to create green and low-carbon buildings, while “Electronic Commerce”, “Agricultural Countryside”, and “New Energy Vehicles” stress on expanding the market of new energy applications to logistics, agriculture, vehicles, etc.
3. Environment-oriented tools: Industrial specifications, standards, and legislation that can make the NEI on the trail are environment-oriented tools, which are embodied in NEP topics with “Science and Education Publicity”, “Marine and Terrestrial Ecology”, and “Regulations and Laws”.

#### 4.1.2 Vertical and horizontal comparisons of policy tools

**Fig 7:**
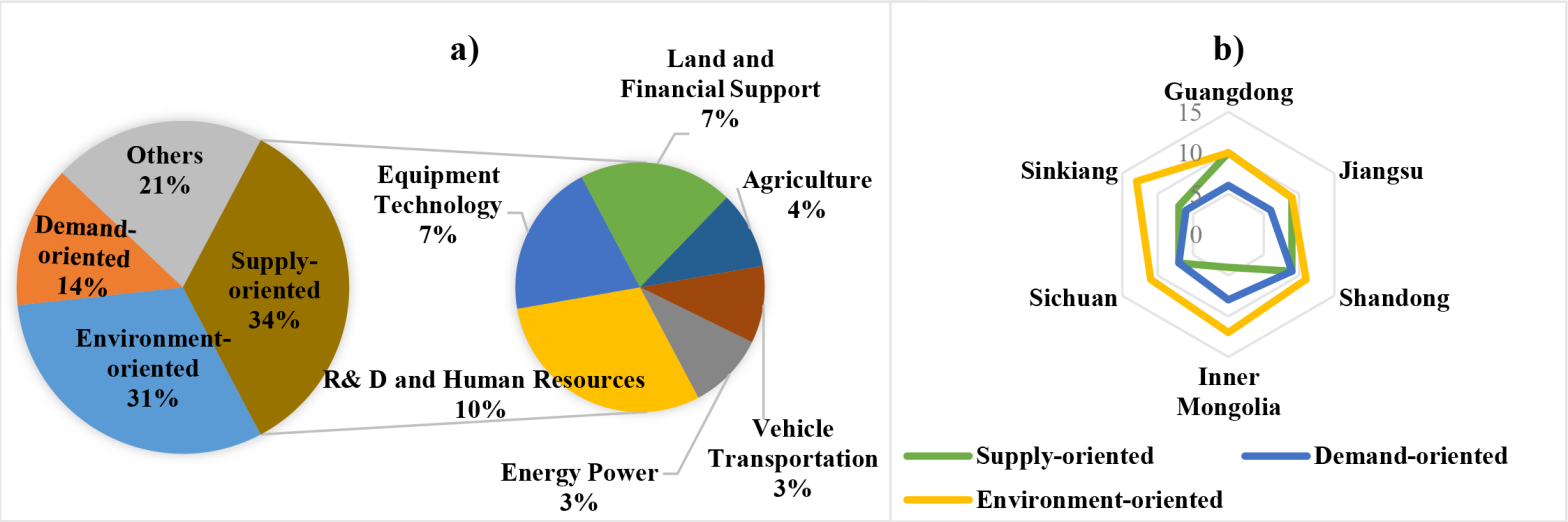
Distribution of policy instruments in topics modeled: a) Central policy tools; b) Provincial policy tools.

Seven topic models have been separately trained on NEP sub-corpora from the central government and six sampled provinces. The distribution of policy instruments is produced by counting the focal topics with different tendencies, as shown in Fig 7.

Subgraph a) shows the policy instruments in central NEP topics, supply-oriented and environment-oriented tools are more commonly used than demand-oriented tools. Moreover, policymakers of the central government emphasize more on talent cultivation, R&D, and financial incentives in the supply-oriented instrument. Subgraph b) displays the preference of provincial policymakers to tool usage. the NEP documents of Sinkiang, Sichuan, and Inner Mongolia put more weight on environment-oriented tools, while Guangdong and Jiangsu provinces pay the same attention to Demand-oriented and Environment-oriented tools. Therefore, since local agencies are branches of the central government, NEP policy choices of Guangdong, Jiangsu, and Shandong are more consistent with those of the central government than the other three provinces, which tend to promote the development environment of NEIs.

### 4.2 NEP topic evolution over time

**Fig 8:**
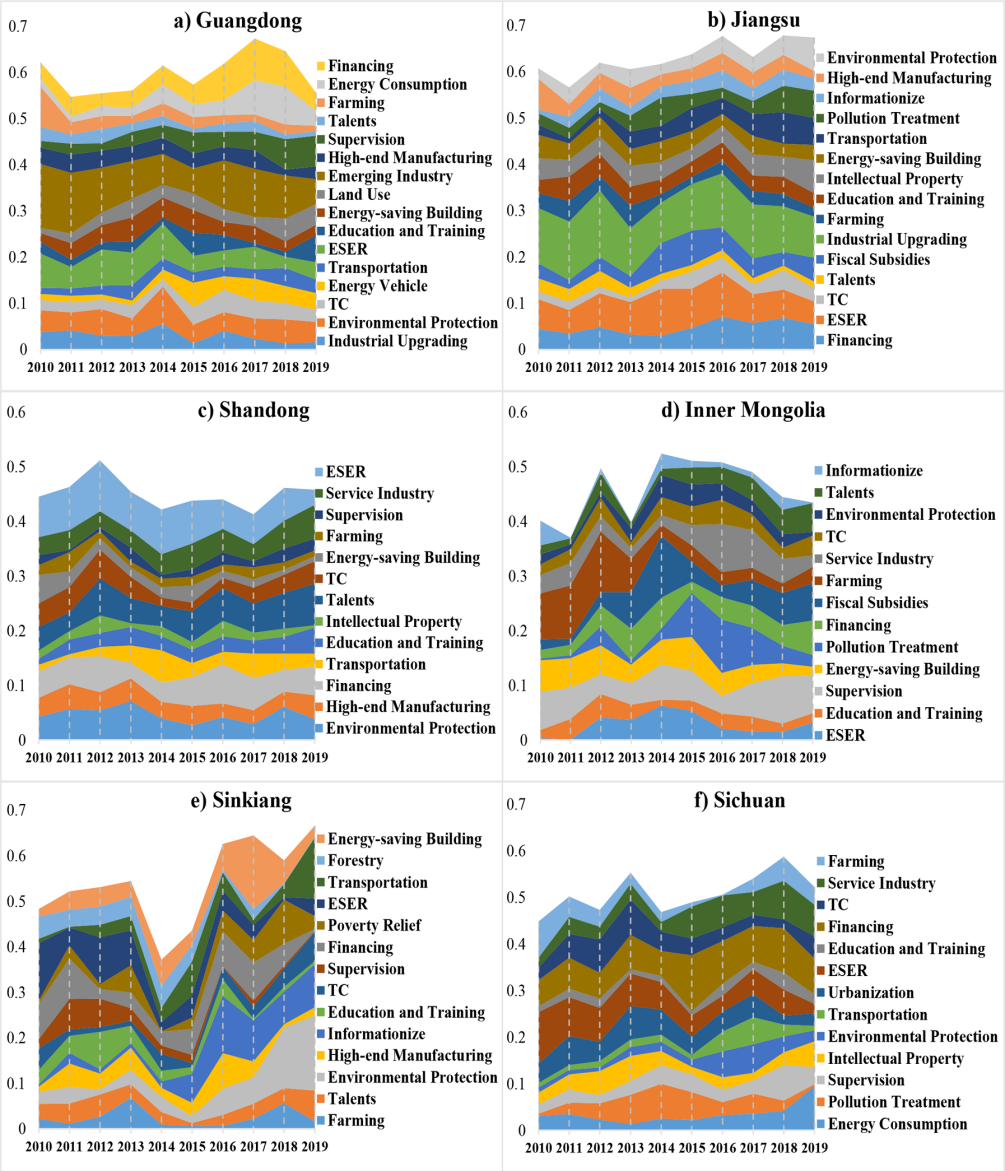
Provincial NEP topic evolutions over time. Some topics with common or unconcerned words are not considered, each topic is named by comprehending its top 10 words.

The static topic model trained in Section 4.1 fails to describe policy topics over time, thus the DTM method is applied to the NEP sub-corpora of sample provinces categorized by publishing time. Fig 8 displays the variations of topic significance in the modeling results. The topic significance, indicating the relative importance of topics obtained from provincial policy texts grouped by timestamps, can be calculated by Formula (2) according to the document-topic matrix that measures the possibility of certain topics appearing in documents [43].

$$Significance_{i}=\sum_{t\in T}p(c_{i}|d_{t})$$ (2)

Where *c*_*i*_ is the *i*-th topic, *d_t_* represents a document in the text group of time *t*(*t* = 1, 2, …,*T*). *Significance*_*i*_ is the topic significance that has been normalized into area bands for comparison in Fig 8.

Compared with Figs 6 and 8 shows the provincial policy topic changes over time, it reflects how provincial governments adjust their focal points of new energy policies over the last decade. Specific dynamics of policy instruments and topics can be illustrated as follows:

1. All subgraphs in Fig 8 get topics “Environmental Protection (EP)”, “Financing”, “Energy Saving and Emission Reduction (ESER)”, “Farming”, and “Education and Training”, which are common concerns in NEPs of the sampled provinces. Guangdong, Jiangsu, and Shandong provinces put more emphasis on energy conservation and environmental protection because the area band corresponding to EP and ESER in subgraphs a), b), c) are wider and more continuous than others. Notably, in recent years, Sinkiang’s government has paid more attention to environmental protection due to the gradual increase of the corresponding area band in subgraph e).
2. Common NEP supply-oriented tools like “Education and Training”, “Financing”, and “Talents” have been widely adopted in provincial policies, but subgraphs a), b) show that policy tools related to “Land Use” and “Fiscal Subsidies” are used by Guangdong and Jiangsu in addition to the traditional tools. Similarly, the terms “Emerging Industry” and “Industrial Upgrading” are the most significant topics of NEP documents in Guangdong and Jiangsu, respectively, which cannot be found in other provincial policies. Therefore, the two provinces with the highest GDP in China provide much financial and manufacturing support to the NEI development.
3. Demand-oriented policy tools like “Trade and Communication (TC)”, “Energy-saving Building”, and “Service Industry” are the most commonly used, in addition to “Electric Vehicles”, “Poverty Relief”, and “Energy Consumption”. From the area charts in Fig 8, the significance of “Service Industry” has increased dramatically in NEPs of Sinkiang and Sichuan provinces in recent years, whereas it rises slower in Guangdong and Shandong provinces. From the area width of topics shown in subgraph d) and e), “Energy-saving Building” has received more attention in the NEPs of Inner Mongolia and Sinkiang. Moreover, Sinkiang and Sichuan also have a unique policy topic on “Poverty Relief”, while Guangdong gets “Electric Vehicles”. Therefore, Inner Mongolia, Sinkiang, and Sichuan provinces prefer to balance the use of various NEI demand-oriented tools in their policies, while Guangdong, Jiangsu, and Shandong stress less on the diversity of these tools due to the already large demand from their economic development.
4. Except for EP and ESER, “Intellectual Property” and “Supervision” topics represent the environment-oriented policy instrument for creating a healthy atmosphere benefit for the NEI development. From subgraph d), in the last decade, the topic strength of supervision represented by the area width has maintained a high level among other sharply changing blocks, indicating that Inner Mongolia province inclines to publish regulations or laws in the NEPs. Although the emphasis on supervision is less than that of Inner Mongolia, it can be seen from subgraphs a) and b) that the significance of either supervision or “Intellectual Property” topic slightly increases over the years, whereas the topic significance of “supervision” in subgraph e) encounters a large landslide from 2011.

### 4.3 Coevolution between policy topics and NEIs

In order to analyze the coevolution between provincial NEPs and NEIs, the correlation analysis of the dynamic policy topics modeled by the DTM and the provincial NEI panel data is employed in this subsection.

#### 4.3.1 Correlation with CO_2_ emission intensity

**Fig 9:**
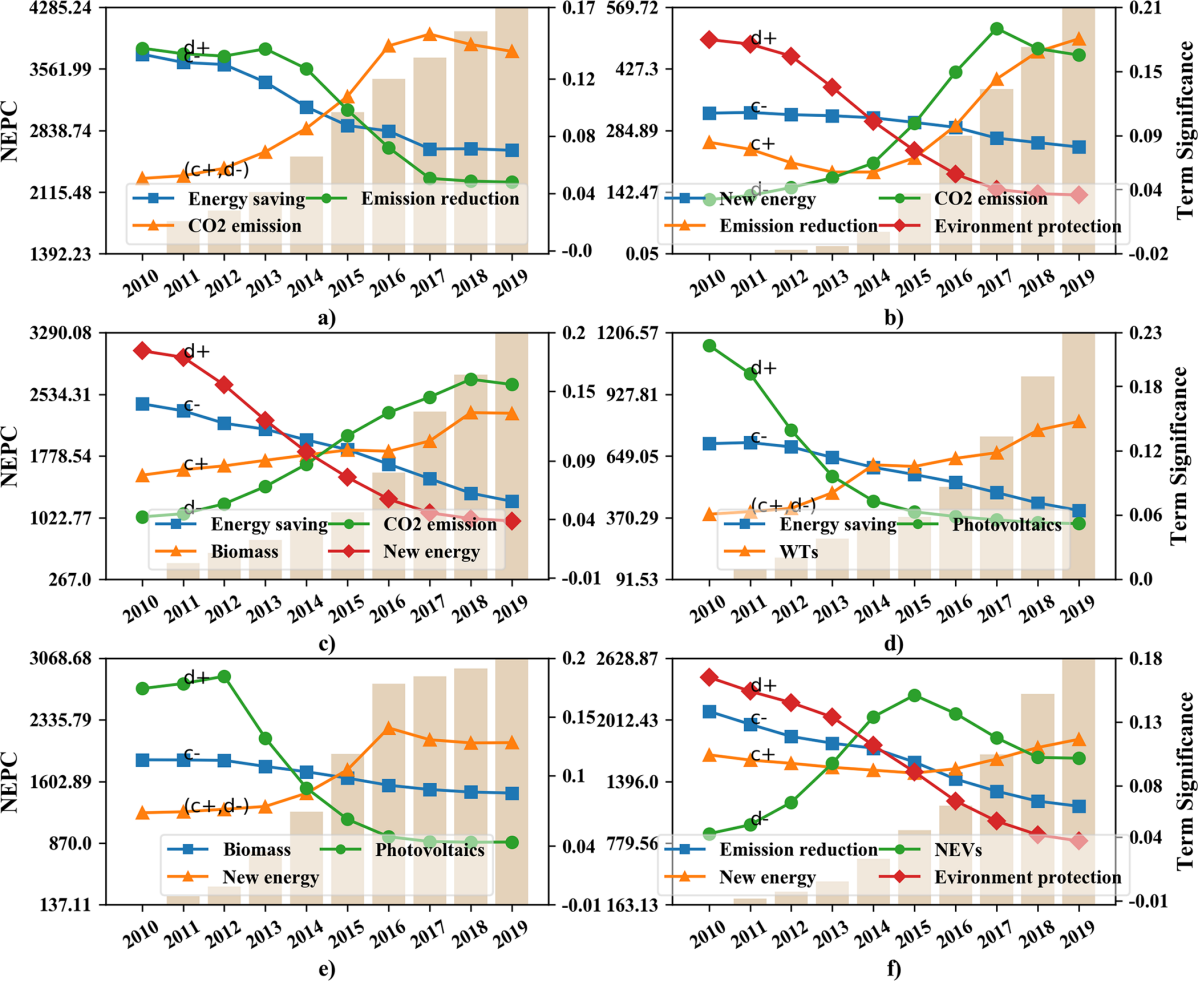
Co-evolution between provincial policy terms and CO_2_ intensity (tons per 10,000 yuan): a) Inner Mongolia; b) Sichuan; c) Shandong; d) Guangdong; e) Sinkiang; f) Jiangsu. c- and c+ represent the minimum and maximum correlations, d- and d+ represent the minimum and maximum DTW distances.

The dynamic time warping (DTW) algorithm is able to find the optimal alignment from two time series, thus is often used to determine time series similarity [44]. Pearson correlation coefficient can be used to measure the strength of the relationship between two variables and their association with each other [45]. In order to investigate underlying relationships between CO_2_ intensity measured by CO_2_ emissions (ton) per 10,000 yuan GDP [46] and policy variations represented by dynamic policy topic terms, both DTW distance and Pearson correlation are used. Fig 9 shows the terms most related to the CO_2_ emission intensity of each province, as well as the most dissimilar and uncorrelated ones (S4 Table). Notably, the CO_2_ emission intensity in subgraphs a) and e) is relatively higher or more abnormal than other subgraphs, which can verify the view mentioned in Sections 4.1.2 and 4.2 that Inner Mongolia and Sinkiang prefer environment-oriented tools such as supervision, environment protection, emission reduction, etc.

The broken lines can be divided into two types (denoted as A and B). A-type lines that are most similar to the trend of CO_2_ intensity are marked with c+ or d- in the figure, while B-type lines with c- or d+ markers are the opposite. Therefore, the intersection of the two is the turning point of the gravity shift of the provincial NEPs.

In subgraphs a) to d), policy terms "Photovoltaic", "New energy", and "Energy saving" often appear in A-type lines, while "CO_2_ emission" often appear in the B-type, indicating that most provinces attach importance to the development of new energy such as PVs at first, and then pay more attention to CO_2_ emission management. However, subgraphs a) and d) get B-type terms "Biomass", while subgraph b) gets "WTs", thus Inner Mongolia, Sichuan, and Guangdong provinces incline to put more stress on new energy resources other than PVs in the most recent years. Moreover, in subgraphs d) and f), the term "NEVs" emerges in their B-type lines, which indicates other trends in the NEPs of Guangdong and Jiangsu.

Notably, subgraph e) is odd since the CO_2_ intensity in it got a significant peak in 2016, and broke lines especially the B-type are flatter than other subgraphs. Hence, the proportion of NEI-related terms change fairly moderate, the effect of Sinkiang NEPs on CO_2_ emission reduction can be unobvious before 2016.

#### 4.3.2 Correlation with the capacity of PVs and WTs

**Fig 10:**
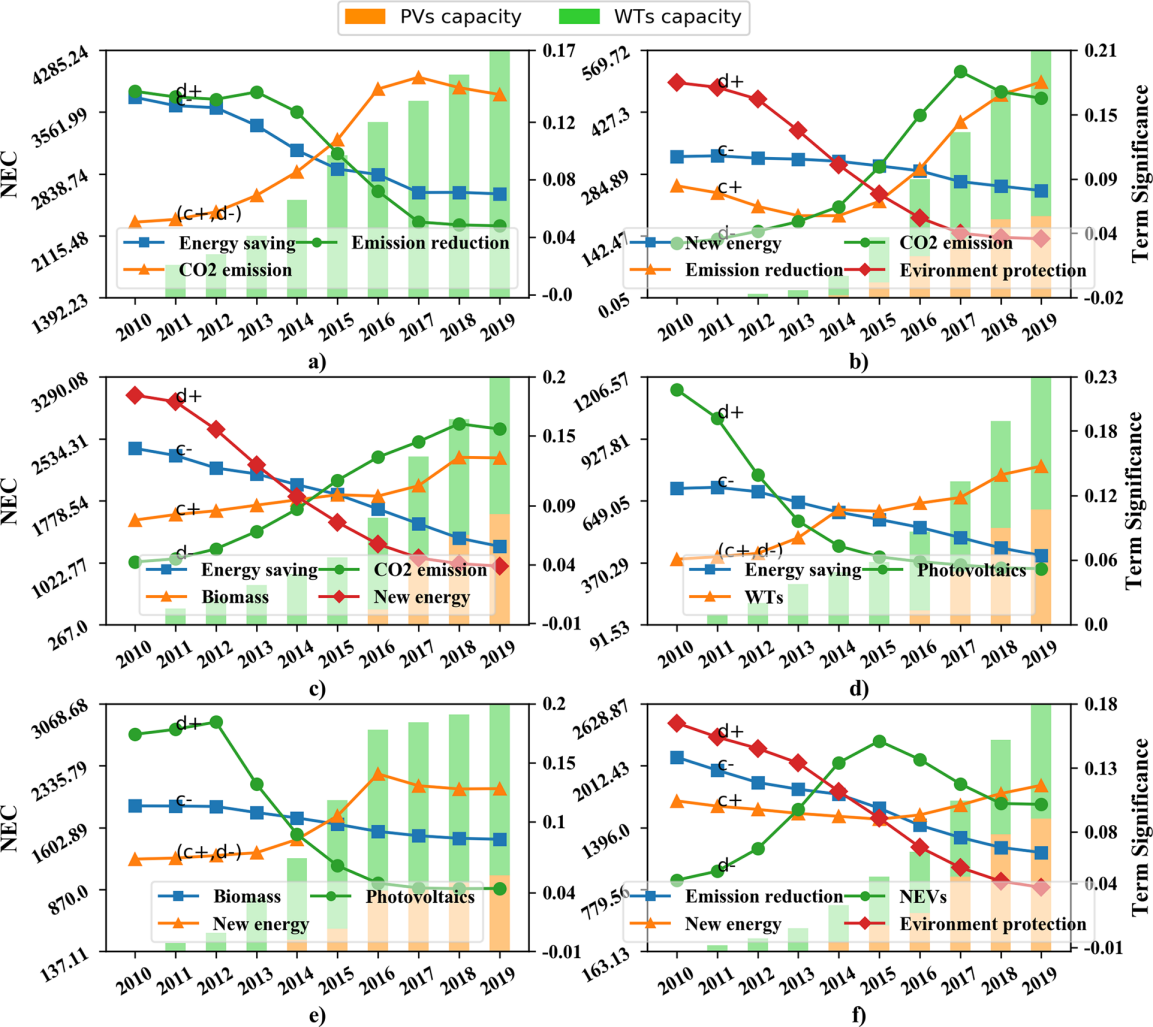
Co-evolution between provincial policy terms and NEC. a) Inner Mongolia; b) Sichuan; c) Shandong; d) Guangdong; e) Sinkiang; f) Jiangsu.

New energy capacity (NEC) is another critical indicator related to the new energy industry, in this paper the capacity of PVs and WTs is considered. Similar to Figs 9 and 10 shows the relationship between the provincial NEC and special NEI terms (S5 Table). It can be seen that the NEC of each province has been growing over the last decade, the installed capacity of wind power is larger than that of solar power in most provinces, but the photovoltaic capacity of Guangdong and Jiangsu gets a tendency to surpass the wind capacity.

The significant hysteretic effects can be observed in Fig 10. For example, although in subgraphs d) and e) the term “Photovoltaics” is highly emphasized in the former half of the subgraphs, the NEC is much lower than it is in the latter part of the subgraphs. Therefore, the opposite trend posed by the NEC and topic terms indicates that the policy expectations and the NEI development are not always synchronized. However, the increase of policy terms "WTs" and "Biomass" are observed in subgraphs a) to d), the NEC can be more direct and synchronized influenced by these terms.

Moreover, Table 3 summarizes the focus shifting process of NEPs during the regional NEI development according to Figs 9 and 10. It can be seen that during the development of regional NEIs, NEPs of most provinces initially focus on “PVs”, “EP”, and “ES” but then transfer to “CO_2_ emissions” and “WTs”. The shifting process usually takes place in about 2014, while it happens earlier in Guangdong and Jiangsu than in other provinces. Recall that in Section 4.1.2 policy tools from NEPs are valued differently by developed and underdeveloped provinces, the discrepancy of the NEP policy focus is more obviously illustrated in Table 3. Developed regions like Guangdong and Jiangsu choose to increase the policy strength of NEVs after about 2014, whereas others including Shandong provinces pay more attention to reduce CO_2_ emissions.

**Table 3:** The shifting policy focus regarding the CO_2_ emission intensity and NEC.

Provinces	The focus before shifting	Transition interval^a^	The focus after shifting	Almost constant
a) Inner Mongolia	PVs, ES, ER	(2014, 2015)	CO_2_ emissions	Biomass
b) Sichuan	PVs, EP	[2014, 2015)	WTs, CO_2_ emissions, ER	NE
c) Shandong	ES, PVs, NE	(2014, 2015)	CO_2_ emissions, Biomass	——
d) Guangdong	NE, PVs, ES	(2013, 2014]	Biomass, NEVs, WTs	——
e) Sinkiang	PVs	[2014, 2015)	NE	WTs, Biomass
f) Jiangsu	WTs, ER, EP	(2013, 2014)	NEVs	NE

^a^ The transition interval is determined by the intersection points of A-type and B-type lines, while the lines corresponding to the last column of the table are not considered.

## 5. Conclusions

In this paper, large-scale NEP texts from central and local governments are modeled by two topic models: (1) the LDA model for extracting NEP topics that are mapped to policy tools; and (2) the DTM for extract time-varying policy topics from provincial NEP documents. The dynamic policy topics are together analyzed with the panel data of provincial NEIs.

Firstly, the NEI of China features regional imbalance to some extent, that is, NEIs are of great potential in the northwest, prosperous in the southeast, and underdeveloped in the southwest. The imbalance is reflected in the various NEP policy instruments embodied by latent topics, as well as the topic evolution paths in a timeline. The most prominent characteristic of the NEP of China is that it incorporates many fields and industries from upstream to downstream, including manufacturing, logistics, agriculture, automobile, construction, etc. Different aspects of NEPs are investigated by the topic generated from policy texts, both central and local governments have paid much attention to the usage of all the policy instruments, namely, the supply-oriented, environment-oriented, and demand-oriented tools. However, the focus of NEPs varies from policymakers of different provinces. Policy topics from NEPs of Guangdong and Jiangsu are more consistent with those from the central policy, which put more weight on supply and environmental policy tools, while other provinces only prefer environmental tools.

Secondly, in spite of the consensus of sample provinces to NEP long-enhanced concerns like environment protection, energy saving, and emission reduction, education and training, and intellectual property, the discrepancy of provincial NEPs over time is closely related to regional geography and economic conditions. Specifically, northwest and southwest provinces with underdeveloped economy incline to adopt measures like poverty alleviation, environmental protection, and supervision, whereas the NEPs of other developed coastal provinces prefer industrial upgrading, NEVs, and emerging industries as time goes by.

Finally, during the correlation analysis of NEPs and NEIs, even Shandong province, the third largest economic province in China, has to shift the focus of NEPs to CO_2_ emission reduction due to its high coal consumption. Therefore, the different evolution paths to NEPs can be mediately explained by the variation of the provincial CO_2_ intensity, which is more unstable or higher in northwest and southwest provinces than others. Moreover, the correlation analysis between the provincial NEC and NEP indicates that some of the policy topic shifts have hysteretic effects on the NEC.

According to the concluded illustrations, this paper advocates the following three points: (1) Backward provinces in China with vast potential in new energy generation should maintain their leading position to provide energy for developed provinces and reduce their CO_2_ emissions by adding more supply-oriented tools to NEPs; (2) All provinces should enhance the demand-oriented policy tools and explore demands for applying new energy resources to the domains other than existing application domains; and (3) When setting the development goals of NEI, policymakers should fully consider the hysteretic effect of policy effectiveness, thus to design policy measures and objectives that are consistent within a certain period.

## Supporting information

S1 Table Provincial NEI performance on the sampling criteria C1-C6. (XLSX)

S2 Table LDA topic modeling results. (XLSX)

S3 Table The evolution of provincial NEP topics modeled by DTM. (XLSX)

S4 Table Relations between carbon dioxide intensity and policy terms. (XLSX)

S5 Table Relations between new energy capacity and policy terms. (XLSX)

## Abbreviations

DTM: Dynamic Topic Model
DRC: Development and Reform Commission
EP: Environmental Protection
ESER: Energy Saving and Emission Reduction
LDA: Latent Dirichlet Allocation
LSA: Latent Semantic Analysis
NEA: National Energy Administration
NEVs: New Energy Vehicles
NEIs: New Energy Industries
NEPs: New Energy Policies
NEC: New energy capacity
PVs: Photovoltaics
RESs: Renewable Energy Sources
TC: Trade and Communication
UHV: Ultra-High Voltage
WTs: Wind Turbines

## References

[1] Andre T. Renewables 2020 Global Status Report. REN 21. 2020 June 16 [Cited 2021 Apr 7]. Available from: https://www.ren21.net/gsr-2020/.

[2] Zou P, Chen Q, Yu Y, Xia Q, Kang C. Electricity markets evolution with the changing generation mix: An empirical analysis based on China 2050 High Renewable Energy Penetration Roadmap. Applied Energy. 2017;185: 56–67.

[3] Liu Q, Lei Q, Xu H, Yuan J. China’s energy revolution strategy into 2030. Resources, Conservation and Recycling. 2018;128: 78–89. 10.1016/j.resconrec.2017.09.028

[4] Jiang X, Green C. China’s future emission reduction challenge and implications for global climate policy. Clim Policy. 2018;18: 889–901. 10.1080/14693062.2017.1388211

[5] Li L, Taeihagh A. An in-depth analysis of the evolution of the policy mix for the sustainable energy transition in China from 1981 to 2020. Applied Energy. 2020;263: 114611. 10.1016/j.apenergy.2020.114611

[6] Liu W, Yi H. What Affects the Diffusion of New Energy Vehicles Financial Subsidy Policy? Evidence from Chinese Cities. IJERPH. 2020;17: 726. 10.3390/ijerph17030726 31979153 PMC7037132

[7] Alizadeh R, Soltanisehat L, Lund PD, Zamanisabzi H. Improving renewable energy policy planning and decision-making through a hybrid MCDM method. Energy Policy. 2020;137: 111174. 10.1016/j.enpol.2019.111174

[8] Heng Y, Lu C-L, Yu L, Gao Z. The heterogeneous preferences for solar energy policies among US households. Energy Policy. 2020;137: 111187. 10.1016/j.enpol.2019.111187

[9] Li W, Long R, Chen H, Dou B, Chen F, Zheng X, et al. Public Preference for Electric Vehicle Incentive Policies in China: A Conjoint Analysis. Int J Environ Res Public Health. 2020;17: 318. 10.3390/ijerph17010318 31906526 PMC6981758

[10] Usman M, Makhdum MSA, Kousar R. Does financial inclusion, renewable and non-renewable energy utilization accelerate ecological footprints and economic growth? Fresh evidence from 15 highest emitting countries. Sustainable Cities and Society. 2020: 102590. 10.1016/j.scs.2020.102590

[11] Danish, Ulucak R, Khan SU-D. Determinants of the ecological footprint: Role of renewable energy, natural resources, and urbanization. Sustainable Cities and Society. 2020;54: 101996. 10.1016/j.scs.2019.101996

[12] Wang Q, Kwan M-P, Fan J, Zhou K, Wang Y-F. A study on the spatial distribution of the renewable energy industries in China and their driving factors. Renewable Energy. 2019;139: 161–75. 10.1016/j.renene.2019.02.063

[13] Pereira IPC, Ferreira FAF, Pereira LF, Govindan K, Meidute-Kavaliauskien I, Correia RJC. A fuzzy cognitive mapping-system dynamics approach to energy-change impacts on the sustainability of small and medium-sized enterprises. J Clean Prod. 2020;256: 120154. 10.1016/j.jclepro.2020.120154

[14] Przychodzen W, Przychodzen J. Determinants of renewable energy production in transition economies: A panel data approach. Energy. 2020;191: 116583. 10.1016/j.energy.2019.116583

[15] Ryan AJ, Donou-Adonsou F, Calkins LN. Subsidizing the sun: The impact of state policies on electricity generated from solar photovoltaic. Econ Anal Policy. 2019;63: 1–10. 10.1016/j.eap.2019.04.012

[16] Dissanayake S, Mahadevan R, Asafu-Adjaye J. Evaluating the efficiency of carbon emissions policies in a large emitting developing country. Energy Policy. 2020;136: UNSP 111080. 10.1016/j.enpol.2019.111053 32675905 PMC7365656

[17] Zhang J, Kang L, Li H, Ballesteros-Pérez P, Skitmore M, Zuo J. The impact of environmental regulations on urban Green innovation efficiency: The case of Xi’an. Sustainable Cities and Society. 2020;57: 102123. 10.1016/j.scs.2020.102123

[18] Hassan ST, Danish, Khan SU-D, Xia E, Fatima H. Role of institutions in correcting environmental pollution: An empirical investigation. Sustainable Cities and Society. 2020;53: 101901. 10.1016/j.scs.2019.101901

[19] Usman O, Alola AA, Sarkodie SA. Assessment of the role of renewable energy consumption and trade policy on environmental degradation using innovation accounting: Evidence from the US. Renew Energy. 2020;150: 266–77. 10.1016/j.renene.2019.12.151

[20] Bento N, Borello M, Gianfrate G. Market-pull policies to promote renewable energy: A quantitative assessment of tendering implementation. J Clean Prod. 2020;248: 119209. 10.1016/j.jclepro.2019.119209

[21] Yi L, Bai N, Yang L, Li Z, Wang F. Evaluation on the effectiveness of China’s pilot carbon market policy. J Clean Prod. 2020;246: 119039. 10.1016/j.jclepro.2019.119039

[22] Lin B, Zhu J. Is the implementation of energy saving and emission reduction policy really effective in Chinese cities? A policy evaluation perspective. J Clean Prod. 2019;220: 1111–20. 10.1016/j.jclepro.2019.02.209

[23] Park C, Yong T. Prospect of Korean nuclear policy change through text mining. Energy Procedia. 2017;128: 72–8. 10.1016/j.egypro.2017.09.017

[24] Proskuryakova LN, Ermolenko GV. The future of Russia’s renewable energy sector: Trends, scenarios and policies. Renewable Energy. 2019;143: 1670–86. 10.1016/j.renene.2019.05.096

[25] Lee JunYeop, Lee Juhyeon. A Text Mining Analysis of US-Chinese Leaders on Trade Policy. JILT. 2019;17: 67–76. 10.24006/jilt.2019.17.3.001

[26] Grazieschi G, Asdrubali F, Guattari C. Neighbourhood sustainability: State of the art, critical review and space-temporal analysis. Sustainable Cities and Society. 2020;63: 102477. 10.1016/j.scs.2020.102477

[27] Lüdering J, Tillmann P. Monetary policy on twitter and asset prices: Evidence from computational text analysis. The North American Journal of Economics and Finance. 2020;51: 100875. 10.1016/j.najef.2018.11.004

[28] Osorio-Arjona J, Horak J, Svoboda R, García-Ruíz Y. Social media semantic perceptions on Madrid Metro system: Using Twitter data to link complaints to space. Sustainable Cities and Society. 2021;64: 102530. 10.1016/j.scs.2020.102530

[29] Han J, Huang Y, Kumar K, Bhattacharya S. Time-Varying Dynamic Topic Model: A Better Tool for Mining Microblogs at a Global Level. J Glob Inf Manag. 2018;26: 104–19. 10.4018/JGIM.2018010106

[30] Huang M, Zolnoori M, Balls-Berry JE, Brockman TA, Patten CA, Yao L. Technological Innovations in Disease Management: Text Mining US Patent Data From 1995 to 2017. J Med Internet Res. 2019;21: e13316. 10.2196/13316 31038462 PMC6611693

[31] Beaudreau BC. On the methodology of energy-GDP Granger causality tests. Energy. 2010;35: 3535–9. 10.1016/j.energy.2010.03.062

[32] Arif M, Ahmad F, Kashyap R, Abdel-Galil TK, Othman MM, El-Amin I, et al. Evaluation of EHV and AC/DC technologies for integration of large-scale renewable generation in Saudi Arabian network. IET Gener Transm Distrib. 2019;13: 575–81. 10.1049/iet-gtd.2018.5229

[33] Van Rossum G. The Python Library Reference, release 3.8.2. Python Software Foundation; 2020.

[34] Bird S, Klein E, Loper E. Natural Language Processing with Python: Analyzing Text with the Natural Language Toolkit. 1st ed. Sebastopol: O’Reilly Media; 2009.

[35] Junyi S. fxsjy/jieba 2020. 2020 Jan 20 [Cited 2021 Apr 7]. Available from: https://github.com/fxsjy/jieba.

[36] Paustian K, Ravindranath NH, Amstel AV. 2006 IPCC Guidelines for National Greenhouse Gas Inventories. 2006 IPCC Guidelines for National Greenhouse Gas Inventories. Volume 4 Agriculture, forestry and other land use; 2006.

[37] National Bureau of Statistics. China energy statistical yearbook. Beijing: China Statistics Press; 2018.

[38] Blei DM, Ng AY, Jordan MI, Lafferty J. Latent Dirichlet Allocation. J Mach Learn Res. 2012;3: 993–1022.

[39] Blei DM, Lafferty JD. Dynamic Topic Models. Proceedings of the 23rd International Conference on Machine Learning, New York, NY, USA: Association for Computing Machinery; 2006, p. 113–20. 10.1145/1143844.1143859

[40] Řehůřek R, Sojka P. Software Framework for Topic Modelling with Large Corpora. University of Malta; 2010.

[41] Röder M, Both A, Hinneburg A. Exploring the Space of Topic Coherence Measures. Proceedings of the Eighth ACM International Conference on Web Search and Data Mining, New York, NY, USA: Association for Computing Machinery; 2015, p. 399–408. 10.1145/2684822.2685324.

[42] Hanson EC, Rothwell R, Zegveld W. Industrial innovation and public policy: preparing for the 1980s and the 1990s. American Political Science Review. 1981;76: 699.

[43] Karpovich S, Smirnov A, Teslya N, Grigorev A. Topic model visualization with IPython. Proceedings of the 20th Conference of Open Innovations Association FRUCT, vol. 776, 2017, p. 131–7. 10.23919/FRUCT.2017.8071303

[44] Berndt DJ, Clifford J. Using Dynamic Time Warping to Find Patterns in Time Series. Proceedings of the 3rd International Conference on Knowledge Discovery and Data Mining, AAAI Press; 1994, p. 359–70.

[45] Benesty J, Chen J, Huang Y, Cohen I. Pearson correlation coefficient. In: Noise reduction in speech processing. Springer; 2009. p. 37–40.

[46] National Bureau of Statistics. China Statistical Yearbook. Beijing: China Statistics Press; 2019.

